# Perinatal exposure to germinated brown rice and its gamma amino-butyric acid-rich extract prevents high fat diet-induced insulin resistance in first generation rat offspring

**DOI:** 10.3402/fnr.v60.30209

**Published:** 2016-02-02

**Authors:** Hadiza Altine Adamu, Mustapha Umar Imam, Der-Jiun Ooi, Norhaizan Mohd Esa, Rozita Rosli, Maznah Ismail

**Affiliations:** 1 Laboratory of Molecular Biomedicine, Institute of Bioscience, Universiti Putra Malaysia, Serdang, Selangor, Malaysia; 2 Department of Nutrition and Dietetics, Universiti Putra Malaysia, Serdang, Selangor, Malaysia; 3 UPM-MAKNA Cancer Research Laboratory, Institute of Bioscience, Universiti Pra Malaysia, Serdang, Selangor, Malaysia

**Keywords:** epigenetics, germinated rice bran, gamma amino-butyric acid, insulin resistance

## Abstract

**Background:**

Evidence suggests perinatal environments influence the risk of developing insulin resistance.

**Objective:**

The present study was aimed at determining the effects of intrauterine exposure to germinated brown rice (GBR) and GBR-derived gamma (γ) aminobutyric acid (GABA) extract on epigenetically mediated high fat diet–induced insulin resistance.

**Design:**

Pregnant Sprague Dawley rats were fed high-fat diet (HFD), HFD+GBR, or HFD+GABA throughout pregnancy until 4 weeks postdelivery. The pups were weighed weekly and maintained on normal pellet until 8 weeks postdelivery. After sacrifice, biochemical markers of obesity and insulin resistance including oral glucose tolerance test, adiponectin, leptin, and retinol binding protein-4 (RBP4) were measured. Hepatic gene expression changes and the global methylation and histone acetylation levels were also evaluated.

**Results:**

Detailed analyses revealed that mothers given GBR and GABA extract, and their offspring had increased adiponectin levels and reduced insulin, homeostasis model assessment of insulin resistance, leptin, oxidative stress, and RBP4 levels, while their hepatic mRNA levels of *GLUT2* and *IPF1* were increased. Furthermore, GBR and GABA extract lowered global DNA methylation levels and modulated H3 and H4 acetylation levels.

**Conclusions:**

These results showed that intrauterine exposure to GBR-influenced metabolic outcomes in offspring of rats with underlying epigenetic changes and transcriptional implications that led to improved glucose homeostasis.

Nutrition plays a vital role in many facets of health, and dietary imbalances are major determinants of chronic diseases such as insulin resistance. Also, chronic diet-related diseases are responsible for more than half of all deaths worldwide and have a great impact on national economies (1). In many industrialized countries today, excess caloric intake is a major determinant of insulin resistance (2). Thus, adequate nutrition is particularly crucial during critical periods in early life (pre and postnatal) because it plays an important role in adult onset of chronic disease (3, 4). Interventions during this critical developmental window might provide opportunities to decrease the burden of metabolic diseases such as insulin resistance later in life (5). It has been well documented that insulin resistance, which underlies the symptomatology in type 2 diabetes mellitus is the key feature of the metabolic syndrome (6).

Diabetes mellitus affects a great majority of people globally, and by 2030 the burden of the disease is estimated to reach 552 million people. The International Diabetes Federation estimated that diabetes mellitus accounts for 5–10% of the total health-care budget in many countries (7). In the absence of a complete cure for this disease, current treatment options seek to keep metabolic derangements under tight control, through one or a combination of lifestyle modifications, medications, and diet (8). Many of the drugs currently in use are expensive, and side effects are of serious concern (9). In this regard, natural products have received heightened interest because of their perceived cost-effectiveness and fewer side effects.

Cereals such as rice and wheat are important foods for their rich carbohydrate content (10). Germination of such cereals is an economical processing technology, with numerous advantages and health benefits (11, 12). Recently, germinated brown rice (GBR) has been seen as one of the most interesting germinated cereal products and it has garnered a great deal of attention, especially in Asian countries (13). During the process of germination the chemical compositions of the rice change drastically because the biochemical activity produces essential compounds and energy for the formation of the seedling. Germination is therefore considered an important way to improve bioactive compounds and health benefits of rice grains, as reported severally (11–13). Among the various bioactive compounds potentiated during germination of rice grains, we have demonstrated that γ-aminobutyric acid (GABA) contributes significantly toward the overall bioactivity of GBR (13). However, the fact that GABA does not explain the overall effects of GBR suggests that multiple bioactive compounds produce the effects of GBR through synergy. In view of the improved glucose and cholesterol homeostasis and other health outcomes observed when GBR is consumed (11–13), we hypothesized that it may have similar effects on offspring of rats when it is consumed during pregnancy. Furthermore, we have demonstrated that *in utero* exposure to brown rice can protect offspring against high fat diet–induced insulin resistance (14). Thus, in the present study, the effects of GBR and its GABA-rich extract were evaluated for their effects on high fat diet–induced epigenetic modifications leading up to insulin resistance in offspring of rats exposed to a high fat–diet in pregnancy.

## Methods

### Chemicals, enzyme-linked immunosorbent assays, and epigenetic kits

All solvents were of analytical grade from Merck (Darmstadt, Germany). Biotechnology grade water was obtained from Sigma-Aldrich (St Loius, MO, USA). Rat adiponectin (Catalog No EZRADP-62K) and insulin (Catalog No EZRMI-13K) enzyme-linked immunosorbent assay (ELISA) kits were procured from Millipore Corporation (Billerica, USA). Rat retinol binding protein-4 dual ELISA kit (Catalog No AG-45A-0012YEK-K101) was obtained from Adipogen International (Liestal, Switzerland). Rat 8-iso prostaglandin (Catalog No E-EL-R2488) and rat leptin ELISA kit (Catalog No E-EL-R0582) were purchased from Elabscience Biotechnology Co., Ltd (Wuhan, P.R.C) and rat interleukin 6 (IL-6) (Catalog No CSB-E04640r) ELISA kit was purchased from Cusabio (Wuhan, P.R.C). MethylFlash™ Methylated DNA Quantification Kit (Fluorometric) (Catalog No P-1035), Epiquick™ Total Histone Extraction Kit (Catalog No OP-0006), Epiquick™ Total Histone H3 Quantification Kit (Fluorometric) (Catalog No P-3063), Epiquick™ Total Histone H4 Quantification Kit (Fluorometric) (Catalog No P-3073), Epiquick™ Total Histone H3 Acetylation Detection Fast Kit (Fluorometric) (Catalog No P-4031), and Epiquick™ Total Histone H4 Acetylation Detection Fast Kit (Fluorometric) (Catalog No P-4033) were obtained from EPIGENETIK (Brooklyn, NY, USA).

### Germination of brown rice and extraction of GABA

Brown rice (MR220 variety) was obtained from PadiBeras Nasional Berhad (Selangor, Malaysia) and germinated as described previously (15). Briefly, 500 g brown rice was washed twice using tap water, after which sodium hypochlorite was added at a ratio of 1:2 (w/v) and left to soak for 30 min. It was then drained and rinsed with distilled water and subsequently soaked in hydrogen peroxide (H_2_O_2_) 1:2 (w/v) prior to incubation for 6 h at 37°C. Then, H_2_O_2_ was drained and the rice incubated once again in a closed plastic container for 18 h at 37°C and later oven-dried at 50°C until moisture content of 8–11% was achieved. It was then ground with a stainless steel grinder (Waring Commercial, Torrington, CT, USA) and used in preparing the rat pellets.

The extraction of GABA from GBR was done according to (15). Ethanol (400 ml; 70%) was mixed with approximately 100 g of ground GBR and sonicated for 30 min. It was then centrifuged for 20 min at 34,800 *g*. Whatman Grade 1 filter paper with the aid of a glass funnel was used to filter the supernatant and the extraction process repeated twice for a total of three extractions. The filtered supernatants were pooled and dried using a rotary evaporator (Rotavapor^®^ R-210, BUCHI, Flawil, Switzerland).

### Experimental design

All animal experiments were implemented in accordance with the guidelines for the use of animals as approved by the Animal Care and Use Committee, Faculty of Medicine and Health Sciences, Universiti Putra Malaysia (approval number UPM/FPSK/PADS/BR-UUH/00360). Fifteen female Sprague Dawley rats raised on regular chow diet by the breeder were fed regular chow *ad libitum* and free water access during the acclimatization week. They were then grouped (*n*=3) into high-fat diet (HFD), regular chow (N), 50% GBR (GBR), high-dose GABA extract (GEHD), and low-dose GABA extract (GELD) (Table 1). Male Sprague Dawley rats that had been fed with regular chow were mated with the female rats, and the female rats were maintained on the above diets during pregnancy and lactation. Food intake was set at 30 kcal/100 g body weight/day for all the groups. After delivery, male offspring were chosen for follow-up on the effects of the perinatal interventions on insulin resistance markers. At week 4, the male offspring (*n*=6 per group) were weaned and maintained on regular chow for 4 weeks. Weekly weights of the offspring were taken for a duration of 8 weeks.

**Table 1:** Animal groups and diets

Groups	High-fat diet^a^ (%)	Others
Normal	0	100% normal rat pellet
High-fat diet	50	50% normal rat pellet
Germinated brown rice	50	50% germinated brown rice
Low-dose GABA extract	100	100 mg/kg/day GABA extract
High-dose GABA extract	100	200 mg/kg/day GABA extract

a High-fat diet was formulated as previously reported by Imam (16).

### Oral glucose tolerance test

In order to appraise the effects of the different treatments in the mothers and offspring on systemic glucose homeostasis, an oral glucose tolerance test was performed. Rats were initially fasted overnight prior to oral glucose tolerance test via intragastric gavage using a glucose solution (2 g/kg body weight). This was done after weaning in the mothers and at 8 weeks postdelivery for the offspring. Blood samples were taken from the tail vein at 0, 30, 60, 90, and 120 min using a glucometer.

### Homeostasis model assessment of insulin resistance (HOMA-IR)

HOMA-IR values were calculated as described by (17)

$$\text{HOMA}-\text{IR}=\frac{\text{Fasting serum glucose(mg/dl)×Fasting serum insulin(μU/ml)}}{\text{2430}}$$

### Insulin, adiponectin, leptin, RBP 4, 8-iso prostaglandin and interleukin 6

Serum levels of these markers were determined using the respective ELISA kits according to the manufacturer's instructions. Absorbance were read on a microplate reader at the recommended wavelengths and the results calculated from the respective standard curves; Insulin (y=0.153x+0.3572, *R*2=0.9911) ng/ml, Adiponectin (y=0.0026x+0.1455, *R*2=0.9985) ng/ml, Leptin (y=.2215x+0.0286, *R*2=0.9982) ng/ml, RBP 4 (y=0.1041x+0.2977, *R*2=0.9875) ng/ml, 8-iso prostaglandin (y=0.0024x+0.3167, *R*2=0.9781) pg/ml, and interleukin 6 (y=0.081x+0.2641, *R*2=0.9804) pg/ml.

### DNA/RNA isolation

DNA and RNA were extracted from liver tissues using the ZR-Duet™ DNA/RNA MiniPrep (Zymo Research, Irvine, CA, USA) according to the manufacturer's instructions. Then, quality and quantity assessments of the extracted samples were done using Implem NanoPhotometer^®^ (Munchen, Germany) prior to storage at −80°C.

### Gene expression analysis

The primers were designed on the NCBI website and purchased from Integrated DNA Technologies (Singapore), while the internal control (*KanR*) was supplied by Beckman Coulter (Miami, FL, USA) (Table 2). Forward and reverse primer stocks were diluted to a concentration of 200 nm and 500 nm, respectively, in nuclease free water. Reverse transcription of each sample RNA (50 ng) along with multiplex universal reverse primers to complimentary DNA (cDNA) was done using an XP Thermal cycler (BIOER Technology, Germany) for 1 min at 48°C, 5 min at 37°C, 60 min at 42°C, 5 min at 95°C, and a final hold at 4°C as stated in the GenomeLab™ Start Kit (Beckman Coulter, Miami, FL, USA). A reaction mixture containing cDNA product (9.3 µl), 5 X PCR Master Mix buffer (4 µl), forward universal primer set mix (200 nM, 2 µl, MgCl2) (25 mM, 4 µl) and Thermo Start Taq DNA polymerase (0.7 µl) (Thermo Fisher Scientific) was then used to run polymerase chain reaction (PCR) in an XP Thermal Cycler (BIOER Technology, Germany). Amplification was done at initial denaturation temperature of 95°C for 10 min followed by 35 two-step cycles of 94°C for 30 sec and 55°C for 30 sec, and lastly at 68°C a single extension cycle for 1 min. Analysis of the PCR products was done by the GeXP machine (Beckman Coulter) which was loaded with a 96-well sample plate containing a mixture of PCR products (1 µl), sample loading solution (38.5 µl), and 0.5 µl DNA size standard 400 (GenomeLab™ GeXP Start Kit; Beckman Coulter). Fragment Analysis module of the GeXP system software was used to analyze the results and subsequently eXpress Profiler software was used for normalization.

**Table 2:** Gene names, accession numbers and sequences of primers for multiplex PCR

Gene description	Accession no.	Primer sequence (with universal tags)
*IPF1*	NM_022852	F: AGGTGACACTATAGAATAACATCTCCCCATACGAAG
		R: GTACGACTCACTATAGGGAAAATAAGAATTCCTTCTCCAG
*B2m*^a^,^b^	NM_012512	F: AGGTGACACTATAGAATAATGCTTGCAGAGTTAAACA
		R: GTACGACTCACTATAGGGATGCATAAAATATTTAAGGTAAGA
*Actb*^a^	NM_031144	F: AGGTGACACTATAGAATAAACTACATTCAATTCCATCA
		R: GTACGACTCACTATAGGGATAAAACGCAGCTCAGTAAC
*GLUT2*	NM_012879	F: AGGTGACACTATAGAATACAGTACATTGCGGACTTC
		R: GTACGACTCACTATAGGGAGACTTTCCTTTGGTTTCTG
*Hprt1*^a^	NM_012583	F: AGGTGACACTATAGAATATCCTCATGGACTGATTATG
		R: GTACGACTCACTATAGGGACTGGTCATTACAGTAGCTCTT
*KanR*^c^		

*IPF1*: insulin promoter factor 1; *B2m*: beta-2-microglobulin; *Actb*: beta actin; *GLUT2*: glucose transporter 2; *Hprt1*: Hypoxanthine-guanine phosphoribosyltransferase; *KanR*: kanamycin resistant.

a Housekeeping genes

b Normalization gene

c Internal control.

### Methylated DNA quantification

Methylated DNA was quantified using a fluorometric kit according to the manufacturers’ instructions. Absorbance was read on a microplate reader at the recommended wavelength and the result expressed as fold change with respect to the HFD result.

### Total histone extraction

Extraction of total histone was done according to the manufacturer's instructions. Quantification of protein was calculated using bovine serum albumin (BSA) as standard curve (y=2.3639−0.0234, *R*^2^=0.9981).

### Total histone H3 and H4 acetylation

Total Histone H3 and H4 Acetylation were performed according to the manufacturers’ instructions. Absorbance were read on a microplate reader at the recommended wavelength and the respective standard curves calculated (y=8.0054x+411.8, *R*^2^=0.9618 and y=835.06x+407.6, *R*^2^=0.9978, respectively). The amount of acetyl histone H3 and H4 were then determined by the formula below:

$$\text{Amount (ng/mg protein)=}\frac{\text{Relative fluorescence units (sample}-\text{blank})\times 1,000}{{\text{Protein (ug)}}^{\text{*}}\times \text{slope}}$$

*Histone extract amount added into the sample well

### Statistical analysis

Data were expressed as mean±standard deviation, and the means were compared using ANOVA (analysis of variance) with Tukey's multiple comparison test. Values for *P<*0.05 were considered statistically significant.

## Results

### Body weight

**Fig. 1:**
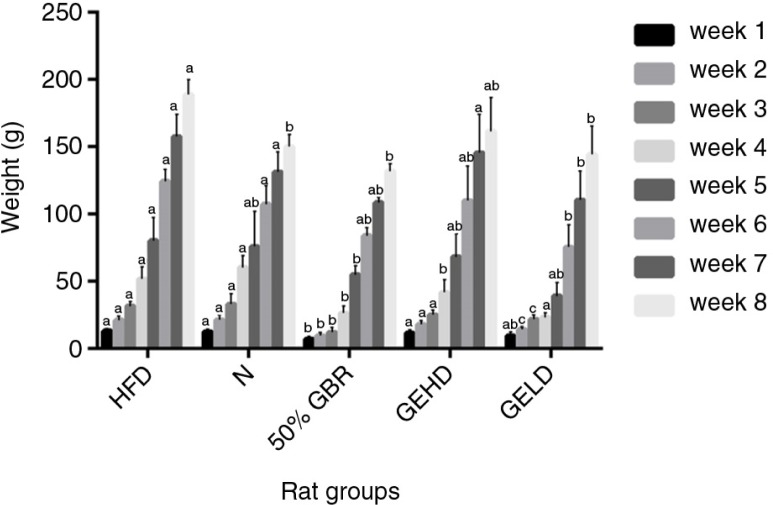
Changes in body weight of offspring (mean±SD, *n*=6) over 8 weeks. Mothers were fed the respective diets for each group, while their offspring were fed regular rat chow after weaning and observed until 8 weeks postdelivery. Abbreviations refer to the diets given to the mothers: high-fat diet (HFD), normal (N), high-fat diet with 50% Germinated Brown Rice (GBR), high-fat diet with GABA-rich extract (gavage) 200 mg/kg body weight (high dose) (GEHD), high-fat diet with GABA-rich extract (gavage) 100 mg/kg body weight (low dose) (GELD). Different letters on bars representing each group indicate statistically significant difference (*p<*0.05).

There were progressive increases in the body weights of the offspring across all groups (Fig. 1). The offspring in the HFD group had the highest weight gain among all groups, whereas those of the 50% GBR group had the lowest weight gain throughout their 8-week postnatal observation and had significantly lower weights at 8 weeks (*p<*0.05) in comparison with the HFD group. The offspring of the GELD and GEHD groups had comparatively similar weight gains throughout the observation period.

### Oral glucose tolerance test

**Fig. 2:**
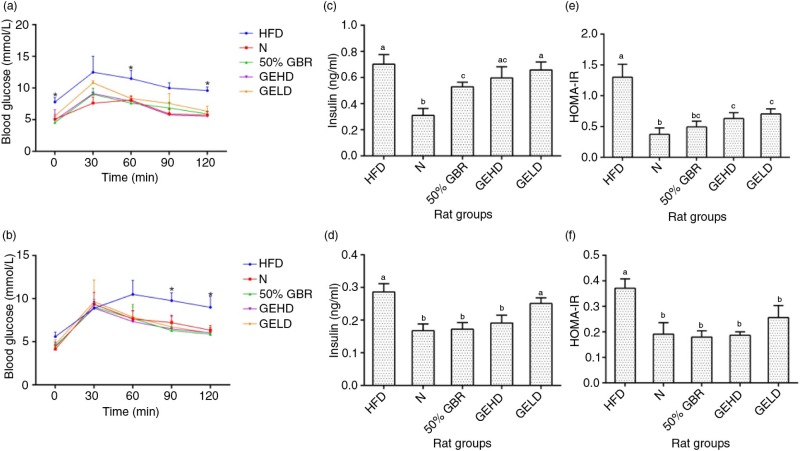
Oral glucose tolerance profiles of (a) mothers and offspring (b). Serum insulin levels of mothers (c) and offspring (d). HOMAR-IR values of mothers (e) and offspring (f). Data for mothers and offspring represent mean±SD (*n*=3) and mean±SD (*n*=6), respectively. Different letters on bars representing each group indicate statistically significant difference (*p<*0.05). Groupings are the same as Fig. 1.

After administration of glucose load in the mothers, blood glucose levels of all the groups, except N, peaked at 30 min (Fig. 2a). Significant differences (*p<*0.05) were observed at 0, 60, and 120 min between HFD and the other groups. At 120 min, the HFD group had the highest blood glucose concentration of 9.60±0.57 mmol/l. No significant differences were observed between GBR, GEHD, and GELD groups at 120 min. In offspring, blood glucose levels of all the groups except HFD, peaked at 30 min. At 90 and 120 min significant differences (*p<*0.05) were observed between HFD and the other groups (Fig. 2b).

### Insulin

Fasting serum insulin levels in the mothers were significantly lower (*p<*0.05) in the GBR group in comparison with the HFD group (Fig. 2c). In offspring (Fig. 2d), fasting serum insulin was significantly higher (*p<*0.05) in the HFD group in comparison with the other groups except GELD.

### Homeostasis model assessment of insulin resistance (HOMA-IR)

The results of HOMA-IR were similar in the mothers (Fig. 2e) and offspring (Fig. 2f). The HFD groups of both mothers and offspring were significantly higher (*p<*0.05) compared with the other groups, which were not significantly different.

### Adiponectin

**Fig. 3:**
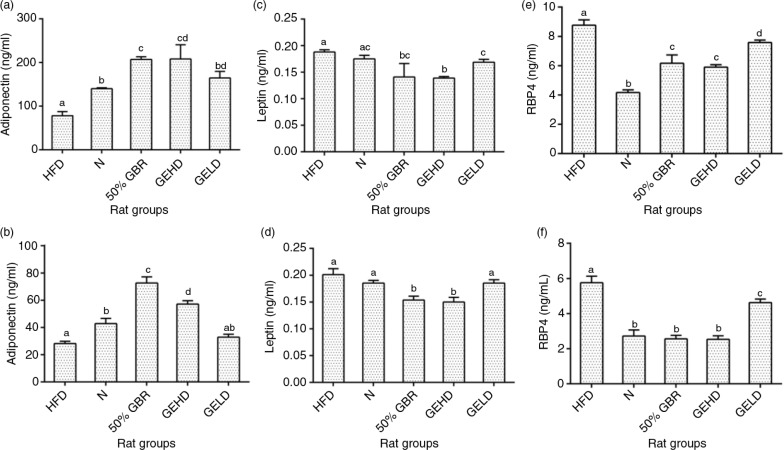
Serum adiponectin levels in mothers (a) and offspring (b). Serum leptin levels in mothers (c) and offspring (d). Serum retinol binding protein 4 (RBP4) levels of mothers (e) and offspring (f). Data for mothers and offspring represent mean±SD (*n*=3) and mean±SD (*n*=6), respectively. Different letters on bars representing each group indicate statistically significant difference (*p<*0.05). Groupings are the same as Fig. 1.

At the end of the intervention, serum adiponectin levels were lowest in the HFD group in the mothers (Fig. 3a), whereas the other groups had significantly higher levels (*p<*0.05). Similarly, the offspring of the HFD group (Fig. 3b) had significantly lower levels in comparison with the other groups, except the GELD group, which was not significantly different.

### Leptin

Serum leptin levels did not differ as much as the other markers. In mothers, the treatment groups had significantly lower levels (*p<*0.05) (Fig. 3c) in comparison with the HFD group. In the offspring (Fig. 3d), a similar pattern was observed in GBR and GEHD.

### Retinol binding protein 4 (RBP4)

Serum RBP4 level was highest in the HFD group, whereas the N group had the lowest level in comparison to the other groups in the mothers (Fig. 3e). In the offspring, a similar pattern was observed although the N, GBR, and GEHD groups had similar levels (Fig. 3f).

### Interleukin 6 (IL-6)

**Fig. 4:**
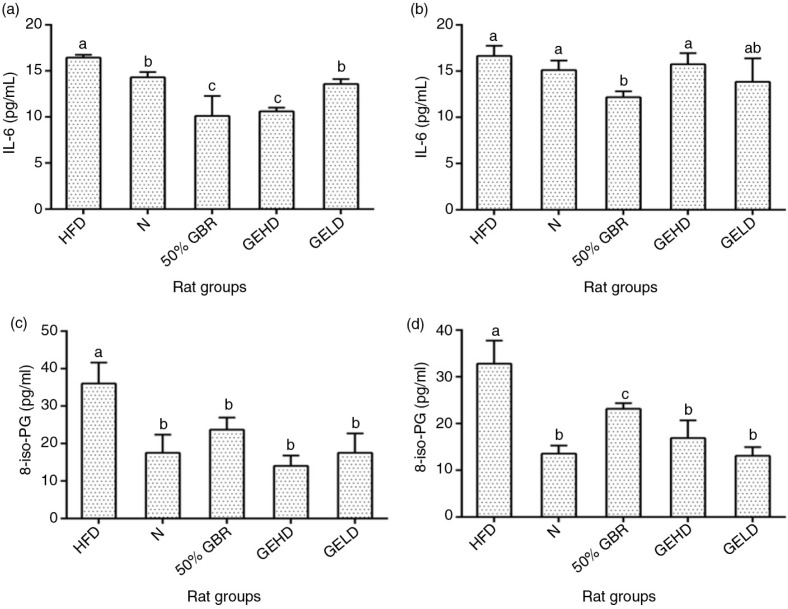
Serum interleukin 6 (IL-6) levels of mothers (a) and offsprings (b), and serum 8-isoprostaglandin (8-iso-PG) levels of mothers (c) and offsprings (d). Data for mothers and offspring represent mean±SD (*n*=3) and mean±SD (*n*=6), respectively. Different letters on bars representing each group indicate statistically significant difference (*p<*0.05). Groupings are the same as Fig. 1.

In mothers (Fig. 4a), serum IL-6 levels were significantly low in GBR and GEHD groups compared to HFD and N and this similarly was observed only in GBR offspring (Fig. 4b).

### 8 – Iso prostaglandin

The 8-isoPG levels in the mothers and offspring had similar patterns, with the HFD groups having the highest levels (*p<*0.05) in comparison with the other groups (Fig. 4c and d).

### Hepatic mRNA levels

**Fig. 5:**
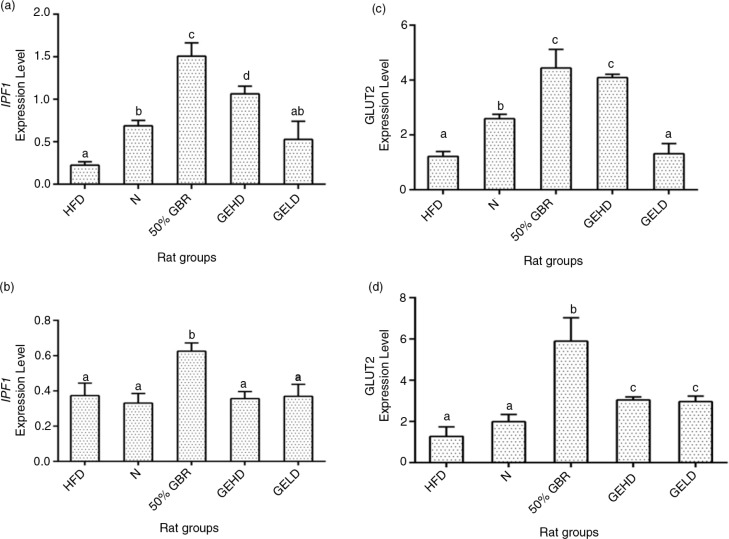
Hepatic mRNA levels of insulin promoter factor-1 (IPF1) in mothers (a) and offsprings (b), and Hepatic mRNA levels of glucose transporter 2 in mothers (c) and offsprings (d). Data for mothers and offsprings represent mean±SD (*n*=3) and mean±SD (*n*=6), respectively. Different letters on bars representing each group indicate statistically significant difference (p<0.05). Groupings are the same as Fig. 1.

The expression of the Insulin Promoter Factor-1 gene was lowest in the HFD group in comparison with the other groups in mothers (Fig. 5a). In offspring, the HFD group showed similar results to those of the N, GELD, and GEHD groups (Fig. 5b), whereas the GBR group had the highest level. Hepatic *GLUT2* expression was lowest in the HFD and GELD groups in the mothers (Fig. 5c), and a similar pattern was seen with the offspring, although in the GELD group it was significantly upregulated compared with the HFD group (Fig. 5d).

### Methylated DNA quantification

**Fig. 6:**
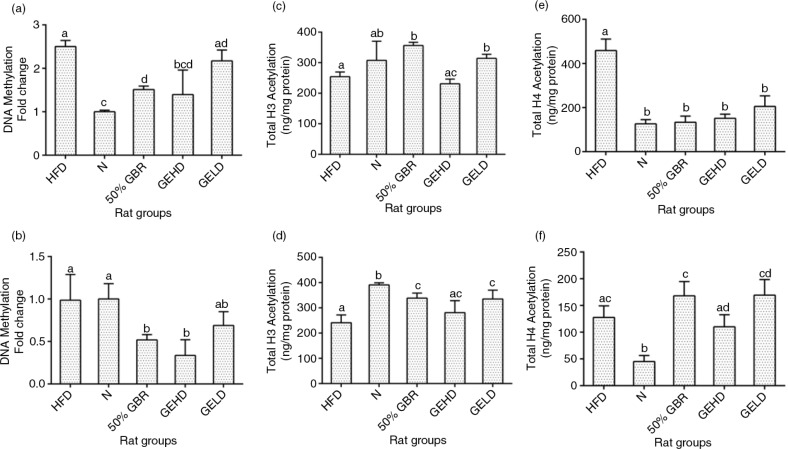
Fold changes in hepatic global DNA methylation in mothers (a) and offspring (b). Hepatic total H3 histone acetylation in mothers (c) and offspring (d). Hepatic total H4 histone acetylation in mothers (e) and offspring (f). Data for mothers and offspring represent mean±SD (*n*=3) and mean±SD (*n*=6), respectively. Data for mothers and offspring represent mean±SD (*n*=3) and mean±SD (*n*=6), respectively. Different letters on bars representing each group indicate statistically significant difference (*p<*0.05). Groupings are the same as Fig. 1.

DNA methylation was expressed in fold change with respect to the N group in both mothers and offspring. In the mothers, the level of DNA methylation was about twofold higher in the HFD and GELD groups compared with the N group, whereas the GBR and GEHD groups had lower levels than in the HFD group (Fig. 6a). Also, in the offspring, the GBR and GEHD groups had significantly lower (*p<*0.05) levels of methylated DNA (Fig. 6b).

### Total histone H3 acetylation

Total histone H3 acetylation (ng/mg protein) levels in the mothers were elevated in the GBR and GELD groups in comparison with the HFD group (Fig. 6c). Acetylation levels were significantly higher in treatment groups in comparison with the HFD groups in the offspring except in the GEHD group (Fig. 6d).

### Total histone H4 acetylation

The H4 acetylation patterns were different from those of the H3. The HFD group had significantly higher (*p<*0.05) levels in comparison with the other groups in the mothers (Fig. 6e), whereas no significant difference was observed when the HFD group is compared with the other treated groups in offspring (Fig. 6f).

## Discussion

A significant number of adults are expected to be overweight and obese by the year 2030 (18). In view of the current global crisis, recent evidence of the complex gene–environment interaction is being closely evaluated as a potential therapeutic avenue for controlling the burden of this pandemic. As can be recalled, adverse prenatal and early postnatal environments influence the risk of developing insulin resistance, type 2 diabetes, and obesity later in life (3, 4). In this study, the body weight changes observed in the offspring in the different groups can be attributed to the different diets they were exposed to *in utero* because they consumed similar amounts of the same regular rat chow after weaning. Previous studies have shown that exposure to HFD *in utero* increases the risk of obesity in offspring (19) similar to our findings. Additionally, we have demonstrated that GBR attenuated weight gain in HFD-fed rats (16), which is similar to what was observed for the mothers in this study. This led to weight regulation in the offspring, and the multiple bioactive compounds in GBR are thought to contribute to this effect as shown by the superior effect of GBR over its GABA-rich extract. Furthermore, we have demonstrated that higher amounts of bioactive compounds in GBR contribute to its superior effects when compared to brown rice (13). The weight regulation by GBR was also expected because we have shown recently that brown rice was able to regulate weights of offspring when their mothers consumed a brown rice diet during pregnancy (14). Insulin sensitivity was assessed by HOMA-IR index. The significantly higher index of insulin resistance HOMA-IR in HFD groups of mothers and offspring demonstrates susceptibility toward an insulin-resistant state, which is associated with the intake of high-fat diets. In contrast, the consumption of GBR and GABA in mothers and by indirect exposure during pregnancy and lactation in offspring allowed for a better glycemic control as seen in lower fasting glucose and insulin levels consequently leading to lower HOMA-IR values.

Furthermore, the development of insulin resistance is closely linked with inflammation and alterations in the secretion of adipocytokines (20). Accordingly, the degree of adiposity has been shown to influence serum leptin adiponectin and IL-6 levels (21). Leptin, by controlling the size of the adipose tissue, regulates weight gain (22), whereas adiponectin enhances metabolism through various mechanisms (23). IL-6 is one of the most studied inflammatory cytokine, which modulates insulin resistance via numerous mechanisms such as the c-Jun N-terminal kinase 1 (*JNK1*)-mediated serine phosphorylation of *IRS1*, with elevated circulating IL-6 levels observed in insulin-resistant subjects (20). In this study, GBR and GABA improved serum leptin, adiponectin, and IL-6 levels toward better metabolic outcomes. Also, RBP4 is an important link between obesity and insulin resistance (24). There is an established relationship between serum RBP4 levels and increased risk of metabolic syndrome of which obesity, dyslipidemia, hyperglycemia, and insulin resistance are a part (25). Similarly, oxidative stress plays a vital role in numerous metabolic abnormalities observed in metabolic syndrome and tends to correlate with RBP4 levels (26). In the present study, HFD-induced metabolic abnormalities in mothers and offspring included elevation of RBP4 and oxidative stress (8-isoPG), which were attenuated by GBR and GABA.

*GLUT2* expression is inversely influenced by HFD (27), in keeping with the present findings. The elevated levels of *GLUT2* expression in the GBR groups are suggestive of enhanced glucose uptake for storage or metabolism. Similarly, *IPF1* downregulation is observed in chronic dyslipidemia and hyperglycemia leading to β cell dysfuction (28). Upregulation of this gene was observed in the GBR and GEHD mothers as well as the GBR offspring. The offspring may have benefited from these effects through intrauterine reprogramming events that tended toward enhanced glucose homeostasis. We therefore hypothesized that epigenetic modifications underlined the effects of the perinatal dietary interventions seen in the offspring. As can be recalled, an adverse intrauterine environment as a result of nutritional imbalances is capable of interrupting the structural and functional programming required in fetal development (5, 6). These are often mediated through epigenetic modification of the genome occurring via DNA methylation or histone modification (4). DNA methylation is essential for normal development and can suppress or activate gene function (29). The most commonly studied histone modifications are those occurring on the lysine residues of H3 and H4 amino termini (30), which have revealed that transcriptional activation of gene is enhanced with increased acetylation (31). In this study, hepatic global DNA methylation, and global acetylation of H3 and H4 histones in the HFD groups were in favor of increased insulin resistance, whereas the GBR and GABA groups produced changes that tended toward improved insulin sensitivity. Multiple factors may have contributed to the epigenetic regulation of the risk of HFD-induced insulin resistance by GBR and GABA, including the potent antioxidative properties of GBR and its constituents (14). Furthermore, oxidative stress can lead to increased DNA methylation with consequent increase in the risk of insulin resistance (32), and attenuation of oxidative stress as seen in this study can lower such risks.

Taken together, the present data showed that GBR enhances metabolic outcomes in HFD-fed rats in keeping with enhanced insulin sensitivity. The data also showed that GBR-derived GABA-rich extract produced some favorable effects (increased adiponectin and reduced leptin, oxidative stress, and RBP4 levels in mothers and offspring). These differential effects may have been because of the multiple bioactive compounds in GBR. The epigenetic effects produced by GBR may have influenced transcriptional regulation of some genes involved in glucose homeostasis in the offspring, which could have been the basis for the changes seen in the GBR offspring. Studies have suggested in the past that nutritional intake does influence the degree of epigenetic modifications (5, 6), and in the present study we showed that GBR exposure during intrauterine development lowers global DNA methylation levels and modulates H3 and H4 acetylation status.

## Conclusions

In the present study, we demonstrated that intrauterine exposure to GBR will influence metabolic outcomes in offspring of HFD-fed rats with underlying epigenetic changes and transcriptional implications that tend toward improved glucose homeostasis. GBR-derived GABA-rich extract also produced favorable metabolic outcomes although not as much as those of GBR. The presence of multiple bioactive compounds in GBR may underlie the superior effects of GBR over its GABA-rich extract. The translational implications of these findings are that consumption of GBR by pregnant women could modulate the risk of insulin resistance and other metabolic perturbations in their offspring even in the presence of environmental factors that promote risk of such diseases through epigenetic modifications. These findings are worth studying further.
